# A Decrease in Effective Renal Perfusion Pressure Is Associated With Increased Acute Kidney Injury in Patients Undergoing Cardiac Surgery

**DOI:** 10.7759/cureus.45036

**Published:** 2023-09-11

**Authors:** Phat T Dang, Balbino E Lopez, Kei Togashi

**Affiliations:** 1 Anesthesiology and Perioperative Medicine, University of California Irvine Health, Orange, USA; 2 Anesthesiology, University of California Irvine Health, Orange, USA; 3 Anesthesiology and Critical Care, University of California Irvine Health, Orange, USA

**Keywords:** intraabdominal hypertension, kidney perfusion pressures, acute kidney injury, intraabdominal pressure, cardiac surgery

## Abstract

Purpose: This study aimed to evaluate the relationship between intra-abdominal pressure (IAP), renal perfusion indices, and postoperative acute kidney injury (AKI) in cardiac patients.

Methods: In a prospective cohort study conducted at a single academic institution, we collected data from adult patients undergoing open-heart operations with cardiopulmonary bypass (CPB) at our institution from February 2022 to April 2022 using the Accuryn SmartFoley system® (Potrero Medical, Hayward, CA). Patients on mechanical support devices, pregnant patients, and patients on hemodialysis were excluded. Demographics, hemodynamics, and mean airway pressure (mAir) were measured at the beginning of the cardiac operations and during the first four hours of ICU. Renal perfusion indices were then calculated (mean perfusion pressure (MPP) = mean arterial pressure (MAP) - central venous pressure (CVP); abdominal perfusion pressure (APP) = MAP - IAP; and effective renal perfusion pressure (eRPP) = MAP - (CVP + mAir + IAP)). Length of stay (LOS) was measured from the day of surgery to ICU discharge (ICU LOS) and hospital discharge (hospital LOS).

Results: During the first four hours of ICU stay, the non-AKI group had lower IAP and higher renal perfusion indices (MPP, APP, and eRPP). Logistic regression showed high perfusion pressures correlated with lower postoperative AKI (all OR <1, p<0.05). The postoperative AKI group also had significantly longer ICU LOS (7.33 vs. 4.57 days) and hospital LOS (17.0 vs. 10.2 days).

Conclusion: Renal perfusion indices are a promising tool to predict postoperative AKI in cardiac surgery patients.

## Introduction

Acute kidney injury (AKI) is one of the major complications following cardiac surgery. While the Society of Thoracic Surgeons National Cardiac Surgery Database reported a postoperative AKI rate of 4%, multiple studies have shown a postoperative AKI rate as high as 81% based on the Kidney Disease Improving Global Outcomes (KDIGO) criteria [1-3]. Most importantly, postoperative AKI, regardless of severity, is associated with increases in major kidney-related morbidities and short- and long-term mortality [1,4]. Following cardiac surgery, features such as inflammatory responses, reperfusion injuries, microembolization of air or atheroma, and hypotension can contribute to the development of AKI [5]. Moreover, the use of cardiopulmonary bypass (CBP) has been shown to increase the risk of postoperative AKI [6]. Renal hypoperfusion, ischemia, and hypoxia are shown to occur at the onset of CPB and continue even after the conclusion of CPB [5]. As a result, cardiac surgery patients face risks of postoperative AKI as high as 81% [6].

Elevation of intra-abdominal pressure (IAP) has been increasingly recognized as an important cause of major morbidities and mortality such as AKI in patients during their ICU stay [7]. Undergoing major abdominal surgery, major burns, or infections such as septic shock or intra-abdominal infections have been shown as risk factors for elevated IAP [8]. Moreover, IAP has been shown to be an independent risk factor for postoperative AKI [7,9,10]. The World Society of the Abdominal Compartment Syndrome further incorporated mean arterial pressure (MAP) and IAP into the abdominal perfusion pressure (APP) to estimate the adequacy of renal perfusion [8]: APP = MAP - IAP. In addition to this, Kopitkó et al. proposed a novel measurement of renal perfusion pressure named “effective renal perfusion pressure (eRPP)” to account for the effects of venous congestion on renal perfusion derived from MAP, IAP, and mean airway pressure (mAir) [11]: eRPP = MAP - (IAP + CVP + mAir). However, these studies were performed in patients undergoing major abdominal surgeries.

Measurement of IAP using a Foley catheter has been previously described. Recently, an FDA-approved foley catheter with the capability of continuously monitoring and measuring IAP, urinary output, and core body temperature has been introduced: Accuryn SmartFoley system® (Potrero Medical, Hayward, CA). The device measures bladder pressure through a balloon containing a pressure sensor at the tip of a Foley catheter and thereby calculates bladder pressure and IAP. Incidences of intra-abdominal hypertension (IAH) in patients undergoing cardiac surgery have been previously reported using this device [12], but the development of AKI in relation to renal perfusion pressure driven by the Accuryn SmartFoley system® has not been reported. Therefore, the aim of this study was to evaluate the relationship between IAP and other renal perfusion indices (APP, eRPP, and MPP) derived from continuously measured bladder pressure and postoperative AKI in cardiac surgery patients. The article was previously posted to the Research Square preprint service on June 6, 2023 [13].

## Materials and methods

Study design and population

We conducted a prospective cohort study of patients undergoing open-heart operations with CPB at our institution from February 2022 to April 2022 using the Accuryn SmartFoley system®, which was capable of continuously monitoring IAP. We obtained approval from the Institutional Review Board at our institution (IRB Protocol#1294) with a waiver of written informed consent. The primary outcome was postoperative AKI, defined by the 2012 KDIGO criteria as at least 0.3 mg/dL increase of serum creatinine level from baseline within 48 hours [14]. The secondary outcome was ICU and hospital length of stay (LOS). Inclusion criteria were age >18 years old and non-transplant cardiac surgery with CPB including isolated coronary artery bypass graft (CABG), isolated single heart valve surgery, multiple heart valve surgery, combined CABG and heart valve surgery, and other non-transplant cardiac surgeries. Exclusion criteria included patients with end-stage renal disease or AKI that required hemodialysis, patients who did not undergo cardiac operations, or patients on preoperative mechanical circulatory support including Impella® (Abiomed, MA, USA) and extracorporeal membrane oxygenation. Cardiac anesthesia management included standard ASA monitors and pulmonary artery pressure, central venous pressure (CVP), and invasive continuous blood pressure monitoring. Accuryn SmartFoley system®, which was capable of continuously monitoring urine output and IAP, was placed at the onset of the surgery to determine IAP in every patient. Postoperatively, patients were sent to the cardiovascular intensive care unit and kept under mechanical ventilation and sedation. During this time period, the patients were kept under moderate to deep sedation (RASS score -3 to -4). Depending on hemodynamic stability and bleeding status, the intensivist determined when to extubate the patients.

Data collection

Demographic information including age, gender, BMI, MAP, CVP, mAir measured by the ventilators, CPB time, and IAP before CPB and during the ICU stay were collected. IAP was collected up to the discontinuation of the Foley catheter. Intraoperative mAir was not available due to the limitation of our anesthesia machine. To assess critical care acuity, EuroSCORE II was calculated for all patients. Since most of our patients underwent fast-track extubation after cardiac surgery, only the first four hours of renal perfusion pressure indices were analyzed. IAP, MPP, APP, and eRPP were collected at baseline (ICU admission) and in one-hour increments. Serum creatinine level and glomerular filtration rate (GFR) were collected preoperatively as well as postoperative day (POD) 1, 2, 3, and on the day of discharge. ICU and hospital LOS were counted from the day of surgery to the day of discharge from either the ICU or the hospital. Multiple renal perfusion pressures and cutoff for high perfusion pressure values as reported by Kopitkó et al. [10,11] were defined in Table 1.

**Table 1:** Definition of renal perfusion variables used in the article mAir was measured by the ventilators when patients were intubated IAP: intra-abdominal pressure, MPP: mean perfusion pressure, APP: abdominal perfusion pressure, eRPP: effective renal perfusion pressure, MAP: mean arterial pressure, CVP: central venous pressure, mAir: mean airway pressure

Variables	Formula	Low cutoff (≤mmHg)
IAP		8
MPP	MAP – CVP	69.5
APP	MAP – IAP	60
eRPP	MAP – (CVP + IAP + mAir)	41

Statistical analysis

Descriptive statistics are presented as number (%) for categorical variables or mean ± standard deviation (SD) for continuous variables. Two-sample Student’s t-test with the assumption of unequal variance (Satterthwaite’s degrees of freedom) was used to assess differences for continuous parametric variables, Mann-Whitney U test for non-parametric variables, and chi-square test for categorical variables as appropriate. We estimated the required sample size to be 20. The sample size was calculated to detect the difference in mean eRPP between 45 mmHg and 30 mmHg with an SD of 10 mmHg to achieve a power of 0.9 with a two-sided alpha level of 0.05. Logistic regression analysis was performed to evaluate the protective effect of high MPP, APP, and eRPP on the odds of developing postoperative AKI as defined by KDIGO criteria. To adjust for possible confounding, Euroscore II (age, gender, presence of chronic pulmonary disease, extracardiac arteriopathy, poor mobility, previous cardiac surgery, active endocarditis, critical preoperative state, renal impairment, diabetes on insulin) and BMI were included in the analysis. All p-values were two-sided, and statistical significance was defined as a p-value of <0.05. All statistical analyses were performed with STATA 18.0 (Stata Corp LP, College Station, TX).

## Results

**Figure 1:**
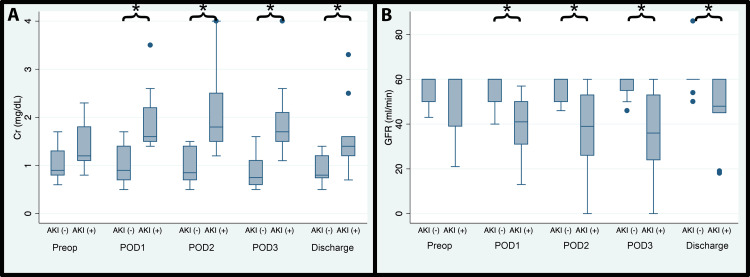
Preoperative and postoperative serum creatinine level (A) and GFR (B) AKI (+): postoperative acute kidney injury, AKI (-): no postoperative acute kidney injury, Cr: creatinine level (mg/dL), GFR: glomerular filtration rate (ml/min), POD: postoperative day. (*) denotes statistical significance (p<0.05)

A total of 23 patients were included in the study. From this, 88 renal perfusion pressure data points were collected. Nine patients (39.1%) had postoperative AKI based on the KDIGO criteria. Most of the patients in the AKI group experienced stage 1 AKI (Table 2). There were no differences between the postoperative AKI and non-AKI in terms of age, gender, weight, BMI, EuroSCORE II, and CBP time (Table 2). Eighteen patients underwent CABG, and five had valvular repair or replacement surgery or a combination of CABG and valvular surgery. The two groups started with similar creatinine levels and GFR at baseline. However, on POD 1, 2, 3, and at discharge, there were significantly higher creatinine levels and lower GFR in the postoperative AKI group (Figure 1).

**Table 2:** Demographic comparison of patients who developed AKI versus non-AKI after cardiac surgery BMI: body mass index, CPB: cardiopulmonary bypass, CABG: coronary artery bypass grafting, EuroScore II: The European System for Cardiac Operative Risk Evaluation II (predicts the risk of in-hospital mortality after cardiac surgery), AKI: acute kidney injury, IAP: intra-abdominal pressure

	Non-AKI	AKI	p-value
Number of patients (%)	14 (60.9)	9 (39.1)	N/A
Age (years)	63.4 (9.83)	67.9 (12.43)	0.371
Weight (kg)	69.5 (21.12)	82.8 (23.56)	0.189
Female (%)	5 (35.7)	2 (22.2)	0.493
BMI (kg/m^2^)	25.3 (6.69)	28.2 (4.62)	0.230
EuroSCORE II (%)	4.8 (5.2)	10.7 (8.8)	0.095
Pre-CPB IAP (mmHg)	7.86 (1.92)	8.17 (1.72)	0.729
CPB time (min)	130.2 (28.82)	135.3 (36.25)	0.726
Preoperative kidney dysfunction (%)	6 (42.9)	5 (55.6)	0.551
Type of surgery			0.524
CABG (%)	10 (71.4)	8 (88.8)	N/A
Single valve (%)	2(14.3)	0 (0.0)	N/A
Multiple valves (%)	1 (7.1)	0 (0.0)	N/A
CABG + valve(s) (%)	1 (7.1)	1 (7.1)	N/A
Stages of postoperative AKI			
Stage 1 (%)	N/A	6 (66.7)	N/A
Stage 2 (%)	N/A	2 (22.2)	N/A
Stage 3 (%)	N/A	1 (11.1)	N/A

Renal perfusion pressure indices

**Figure 2:**
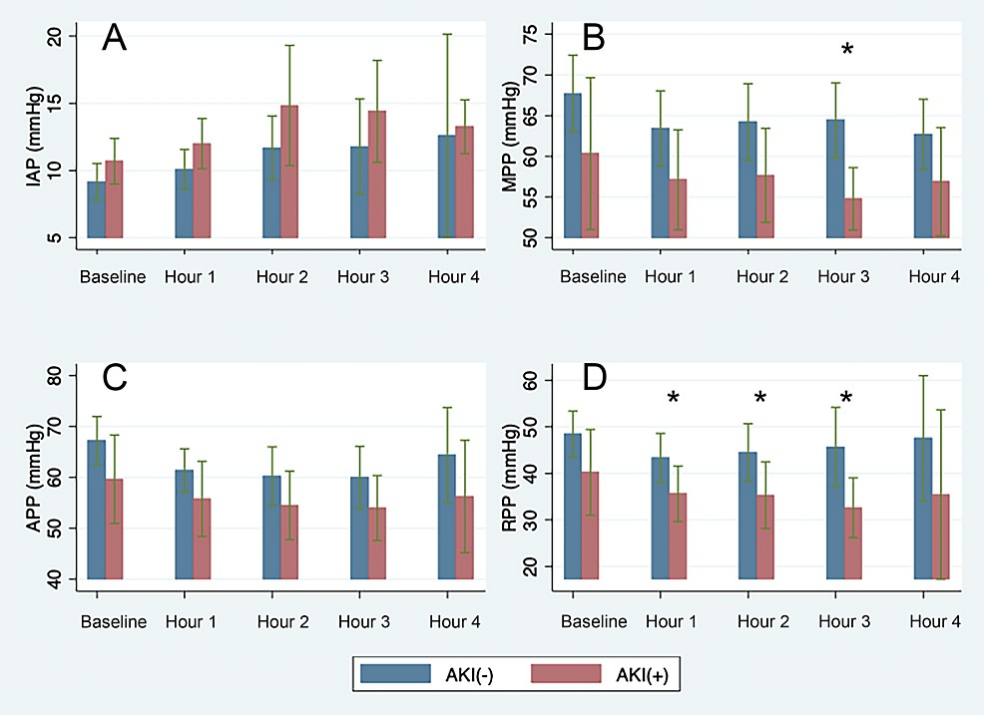
Differences in IAP and renal perfusion pressures between the postoperative AKI group (AKI (+)) and non-AKI group (AKI (-)) AKI (+): postoperative acute kidney injury, AKI (-): no postoperative acute kidney injury. (A) IAP comparison between baseline (at ICU admission) and ICU hours one to four shows higher IAP in the AKI (+) group. (B, C, and D) Comparison between MPP (B), APP (C), and eRPP (D) between AKI (+) and AKI (-) at baseline (ICU admission) and ICU hours one to four. The figures show higher renal perfusion pressures in the AKI (-) compared to AKI (+) group throughout the first four hours of ICU stay. (*) denotes statistical significance (p<0.05)

Initial IAP before CPB were normal and not significantly different between non-AKI and AKI groups (7.86 (95%CI: 4.02, 11.70) vs. 8.17 mmHg (95%CI: 4.73, 11.61), respectively). Furthermore, IAP and kidney perfusion indices (MPP, APP, and eRPP) were not significantly different at ICU admission (Figure 2). However, during the first four hours of ICU stay, there were notable differences between the two groups. Overall, IAP was higher, and renal perfusion indices were lower in the AKI group (Figure 2). On average, IAP was 1.93 mmHg ((95%CI: 0.48, 3.39), p=0.01) higher in the postoperative AKI group in the first four hours of the postoperative period. Similarly, in the first four hours after surgery, the non-AKI group had higher average renal perfusion indices: 7.14 mmHg ((95%CI: 2.03, 12.26), p=0.09) for MPP, 6.41 mmHg ((95%CI:1.18, 11.63), p=0.019) for APP, and 8.92 mmHg ((95%CI: 3.44, 14.41), p=0.03) for eRPP. Interestingly, eRPP was the only index to have consistently higher perfusion pressure even on an hourly basis for the first three hours of ICU admission for the non-AKI group. AKI group also had a longer LOS: 2.76 days ((95%CI: 0.80, 4.72), p<0.001] for ICU LOS and 6.79 days ((95%CI:3.23, 10.35), p<0.001) for hospital LOS.

Effect of high renal perfusion pressure

Logistic regression analysis revealed that high perfusion pressures as defined above significantly correlated with lower odds of postoperative AKI development (Table 3).

**Table 3:** Odds Ratio of high renal perfusion pressures on postoperative AKI MPP: mean perfusion pressure, APP: abdominal perfusion pressure, eRPP: effective renal perfusion pressure

	Odds ratio	95% CI	p-value
MPP	0.22	0.06-0.79	0.02
APP	0.37	0.15-0.96	0.04
eRPP	0.30	0.12-0.76	0.01

## Discussion

This was the first study to investigate the effect of renal perfusion pressure indices on AKI for patients undergoing cardiac surgery with CPB. We assessed renal perfusion pressure values for 23 patients across 88 time points and observed the negative effect of lower renal perfusion pressure on postoperative kidney function (Figure 2). The incidence of postoperative AKI (39.1%) was comparable to other studies [15]. We incorporated a novel Foley catheter with the capability of continuously monitoring and measuring IAP, urinary output, and core body temperature (Accuryn SmartFoley system®). Together with the mAir value driven from the ventilator, we were able to capture an accurate estimate of continuously monitored renal perfusion indices including eRPP.

Increased IAP has been previously shown to increase rates of renal failure in critically ill patients, both surgical and medical patients [8]. In patients undergoing major abdominal surgery, Kopitkó et al. further elucidated this concept by proving that IAP, CVP, and mAir during intubation periods could cause renal venous congestion and the incidence of postoperative AKI [10,11]. In their studies, a novel renal perfusion index, eRPP, which includes MAP, CVP, IAP, and mAir above 40.7 mmHg, was revealed to be protective against postoperative AKI.

We observed a significantly higher IAP in patients who developed postoperative AKI compared to patients without AKI in the first four hours after ICU admission, confirming the observation in medical ICU patients with renal failure (IAP >12 mmHg) [9]. In addition to this, high MPP, APP, and eRPP were also shown to significantly decrease the odds of postoperative AKI (Table 3). Among the indices, eRPP was significantly higher even on an hourly basis (hours one to three after ICU admission) for the non-AKI group which may represent the sensitive nature as a prognostic indicator of AKI development. The most notable observation from our study was that when patients experienced poor renal perfusion pressure as early as the first four hours after ICU admission, it was related to an increase in serum creatinine from baseline (Figure 1). It is worth noting that this may have had a lasting effect leading to increased ICU and hospital LOS in our study.

We posit renal injury in cardiac surgery to be a multifactorial process (intraoperative “hit” and postoperative “hit”), similar to the Knudson two-hit theory for cancer genetics [16]. The initial insult is unavoidable in these cases, as CBP and its associated effects on hemodynamics and inflammatory cascades are necessary in many cardiac surgeries. During CPB, the kidneys, especially the renal medulla, are susceptible to hypoxia due to multiple reasons. First, most CPBs employ hemodilution, thus causing a decrease in oxygen-carrying capacity. Haase et al. showed that the decrease in hemoglobin level during CPB was an independent risk factor for postoperative AKI [17]. Moreover, CPB is associated with inflammation and oxidative stress, which is detrimental to renal perfusion by vasoconstriction of the renal vessels. Loss of pulsatility during CPB is also harmful to the kidneys, resulting in renal vasoconstriction [6,18,19]. Microembolism of gas and particulate also contributes to postoperative renal dysfunction [19]. The second “hit,” which can be thought of as reduced renal perfusion after CPB, however, is where intervention can be used in the ICU post-op. Increased IAP is common in postcardiac surgery patients. Dalfino et al. [9] and Iyer et al. [20] showed an incidence of 31.6-46% of IAH. It has been shown to increase rates of renal failure in postcardiac surgery patients. However, there was no study utilizing the renal perfusion indices in predicting postoperative AKI.

Our study was limited by the observational nature of the study design. Therefore, the results can only infer correlation and not causation. In addition, since eRPP includes mAir, we were not able to monitor this once the patient was extubated. For this reason, since most of our patients underwent fast-track extubation postoperatively, we only analyzed renal perfusion pressure indices up to four hours after ICU admission. A small subset of patients experienced prolonged intubation due to hemodynamic instability or coagulopathy. However, a comparison between this subset of patients and the early extubated patients after four hours would not be inappropriate. Despite this limitation, we still observed a positive correlation between low renal perfusion pressure and the development of AKI. These findings persisted after adjusting for potential confounders using logistic regression analysis. Although AKI and non-AKI groups did not differ in renal perfusion pressure at the time of ICU admission, we were not able to measure renal perfusion pressure indices during surgery. Therefore, we cannot assess the influence of intraoperative renal perfusion pressure on postoperative AKI development. Further research into AKI prevention using composite renal perfusion indices encompassing a wider time span would be beneficial for this patient population.

## Conclusions

Composite renal perfusion indices such as eRPP, starting from the first four hours after surgery, had a significant relationship with postoperative AKI development for patients undergoing fast-track postoperative care after routine cardiac surgery. Future studies using a randomized control design with a longer time span can help establish the causation between renal perfusion pressure and the development of postoperative AKI.
